# Retrospective single-arm cohort study of video-assisted thoracic surgery for treatment of idiopathic peripherally located simple type pulmonary arteriovenous malformation in 23 consecutive patients

**DOI:** 10.1186/s13019-023-02335-w

**Published:** 2023-07-04

**Authors:** Toshiyuki Irie, Osamu Ishibashi, Masashi Kuramochi, Hideo Ichimura, Katsuyuki Endo

**Affiliations:** 1 Department of Radiology, Tsukuba University Hospital Mito Clinical Education and Training Center, Mito Kyodo General Hospital, Miyamachi 3-2-7, Mito City, 310-0015 Ibaraki prefecture Japan; 2 Department of Thoracic Surgery, Tsukuba University Hospital Mito Clinical Education and Training Center, Mito Kyodo General Hospital, Mito City, Japan; 3 Department of Radiology, Hitachi General Hospital, Hitachi City, Japan; 4 Department of Thoracic Surgery, Hitachi General Hospital, Hitachi City, Japan

**Keywords:** Simple type pulmonary arteriovenous malformation, Transcatheter, Embolization, Video-assisted thoracic surgery, Pulmonary wedge resection

## Abstract

**Background:**

Although case reports of video-assisted thoracic surgery (VATS) for pulmonary arteriovenous malformation (PAVM) have been published, studies analyzing more than 10 cases were limited. A retrospective single-arm cohort study was performed to investigate the efficacy of VATS in 23 consecutive patients with idiopathic peripherally located simple type PAVM.

**Methods:**

VATS was performed for wedge resection of 24 PAVMs in 23 patients, which included 4 males and 19 females with an age range of 25 to 80 years (mean: 59.6 ± 13.0). Two patients underwent simultaneous resection of lung carcinoma, one by wedge resection and another by lobectomy. Each medical record was analyzed according to the resected specimen, bleeding volume, postsurgical hospital stay length, duration of chest tube placement, and VATS time. The distance between pleural surface/fissure and PAVM was measured on CT, and the influence of this distance on identification of PAVM was investigated.

**Results:**

In all 23 patients, VATS was successfully performed, and the venous sac was included in each resected specimen. Bleeding volume was less than 10mL in all but one with 1900 mL bleeding volume due to simultaneous lobectomy for carcinoma, not wedge resection of PAVM. Postsurgical hospital stay length, duration of chest tube placement, and VATS time were 5.0 ± 1.4 days, 2.7 ± 0.7 days, and 49.3 ± 39.9 min, respectively. In 21 PAVMs with a distance of 1 mm or less, purple vessel or pleural bulge of PAVM was identified soon after insertion of a thoracoscope. In the remaining 3 PAVMs with a distance of 2.5 mm or more, additional efforts were needed for identification.

**Conclusion:**

VATS was found to be a safe and effective to treatment for idiopathic peripherally located simple type PAVM. When the distance between pleural surface/fissure and PAVM was 2.5 mm or more, a plan and strategy for identification of PAVM should be prepared before VATS.

## Background

Despite the first report of open thoracotomy resection of pulmonary arteriovenous malformation (PAVM) in 1942 [1], the advent of transcatheter embolization (TCE) in 1977 and 1978 [2, 3] has resulted in its becoming the first-line treatment for this condition, given its minimally invasive nature as compared to open thoracotomy. Subsequently, minimally invasive video-assisted thoracic surgery (VATS) was developed, but its use remains limited in comparison to TCE, which is still the sole first-line treatment [4]. However, TCE requires exposure to irradiation, and persistent PAVM may be observed even after initially successful TCE [5]. In contrast, VATS is a definitive treatment that eliminates persistent PAVM after successful resection. Although case reports on VATS for PAVM resection have been published [6–11], studies analyzing 10 or more cases have been limited [12] to the best of our knowledge. Given the benefits and drawbacks of VATS and TCE, our two institutions have employed VATS for the treatment of peripherally located PAVM. The objectives of this paper are to retrospectively analyze consecutive cases and discuss the role of VATS in the treatment of PAVM.

## Materials and methods

The study protocol was a retrospective design approved by the Institutional Review Board (IRB 22 − 18) of Mito Kyodo General Hospital and Hitachi General Hospital (IRB 2022-86), following the ethical principles for medical research involving human subjects as described in the Declaration of Helsinki. The Review Board of Mito Kyodo General Hospital and the Review Board of Hitachi General Hospital waived the informed consent for disclosure of this study due to its retrospective nature and minimal expected risk to subjects. The selection of these two hospitals was based on the author’s previous experience of working at Hitachi General Hospital and current affiliation with Mito Kyodo Hospital.

### Patients

The treatment method for patients with PAVM was selected primarily based on the findings of computed tomography (CT) imaging, and either VATS or TCE were employed. The diagnosis of PAVM before surgery was based on enhanced thin slice CT images that revealed a tortuous tubular structure connecting both the pulmonary artery and vein. Peripheral location of PAVM made it easier to identify it on thoracoscopy and was a factor for selecting VATS. However, pleural thickening/calcification indicating pleural adhesion was a factor to avoid VATS. No strict threshold for each factor was established, and the final decision for treatment method was made through a discussion between the surgeons and interventional radiologists.

In October 2021, medical records of 23 patients who underwent wedge resection of 24 peripherally located simple type PAVMs were accessed from both hospitals. VATS was performed during the period between 2006 and 2020, and informed consent for VATS was obtained from each patient. Patients’ ages ranged between 25 and 80 years old (mean: 59.6 ± 13.0), and there were 4 males and 19 females. One patient had 2 PAVMs in the right lung, and both were resected simultaneously. The remaining 22 patients had a single PAVM.

Spirometry test was within normal limits, and performance status was 0 in all. No patient exhibited clinical symptoms such as dyspnea, cyanosis, or clubbing fingers, except for one patient who showed brain abscess, which led to the detection of PAVM on CT. In 19 patients, PAVM was detected on surveillance CT of primary lung carcinoma. In two patients with colon carcinoma, PAVM was detected on CT to check lung metastases. In one patient, PAVM was incidentally detected in the basal segment on abdominal CT to examine the cause of abdominal pain.

Six patients were with carcinoma. Two patients had lung carcinoma in the same side lung with PAVM and underwent simultaneous resection; one by wedge resection and another by lobectomy. Two patients had lung carcinoma in the contralateral lung and underwent resection of lung carcinoma 2 months after PAVM resection and 8 months before PAVM resection, respectively. Two patients underwent resection of colon carcinoma 19 days before PAVM resection and 9 days after PAVM resection, respectively. No patient had hereditary hemorrhagic telangiectasia (HHT), and the cause of PAVM was idiopathic in all cases.

During the same period, two patients were treated by open thoracotomy: one with complex type PAVM and one with simple type PAVM and lung carcinoma. One patient with complex type PAVM was treated by VATS, and two patients with peripherally located simple type PAVM were treated by VATS, but their medical records were incomplete. These five patients were not included in the analysis, although all of them were successfully treated.

We were prepared to conduct TCE during the same timeframe, however, the absence of patients with centrally located PAVM (e.g., the distance between the pleural surface/fissure and PAVM was 10 mm or more), as well as those with pleural thickening/calcification, resulted in no TCE procedures being performed.

### VATS procedures

**Fig. 1:**
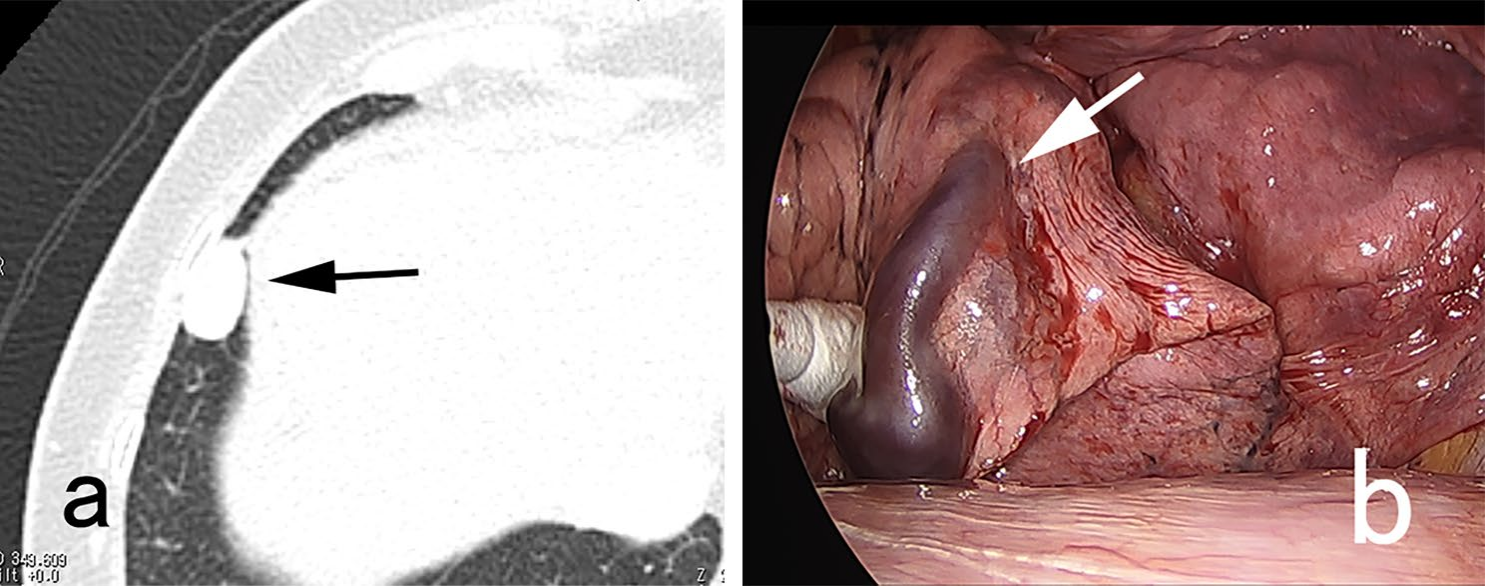
A case with visible PAVM. The distance between pleural surface and PAVM (**a, arrow**) was 0 mm, and purple vessel (**arrow, b**) was seen through the pleura

**Fig. 2:**
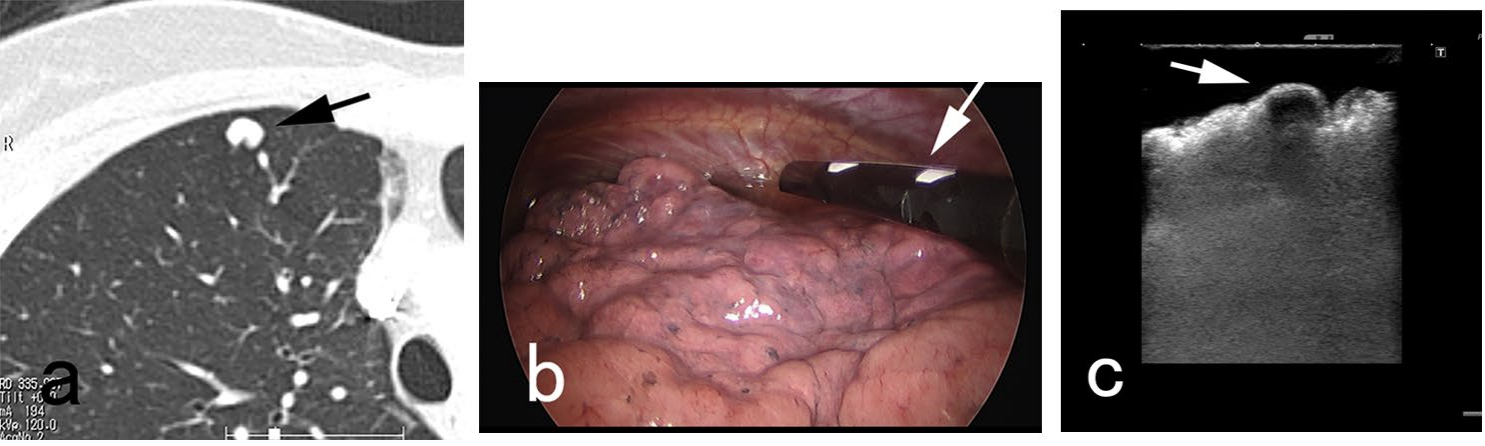
A case with invisible PAVM without pleural bulge. The distance between pleural surface and PAVM (**arrow, a**) was 2.5 mm on CT, and no purple vessel nor pleural bulge of PAVM were seen. Intraoperative ultrasonography (**arrow, b**) well depicted the PAVM (**arrow, c**)

**Fig. 3:**
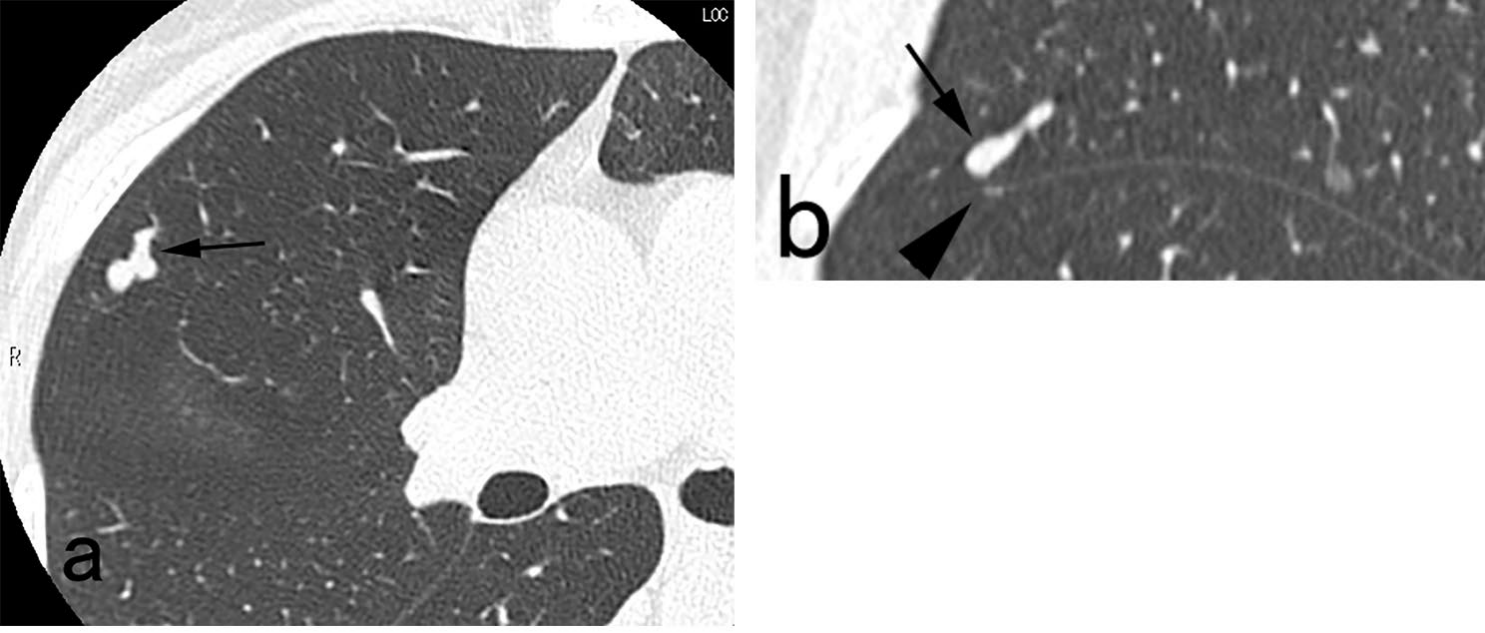
A case with invisible PAVM without pleural bulge. The distance between pleural surface and PAVM (**arrow, a**) was 4.6 mm. Reformatted sagittal CT image clearly depicted the relationship between the PAVM (**arrow, a**) and the margin of the incomplete minor fissure (**arrowhead, b**). The surgeon promptly detected the PAVM soon after making incision into the incomplete minor fissure, and accomplished wedge resection of the PAVM within 24 min

**Fig. 4:**
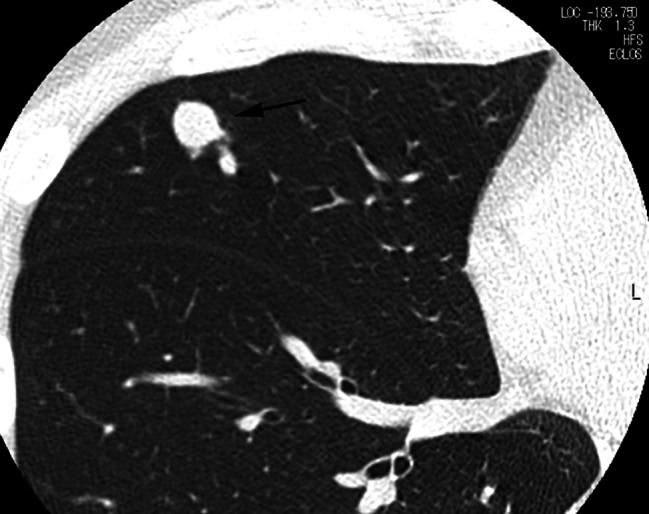
A case with invisible PAVM without pleural bulge. Although the diameter of the venous sac was 11 mm and relatively large, the PAVM, located 5.5 mm below the pleural surface, could not be identified immediately after insertion of the thoracoscope. The ipsilateral lung spontaneously contracted excessively while searching the PAVM, and the surgeon could identify the pleural bulge of PAVM approximately 40 min later

A three-port VATS approach was utilized for wedge resection of each PAVM. Two small ports with skin incisions measuring 1 cm in length were created for the insertion of a thoracoscope and forceps. A third port with a skin incision of 2 cm or greater was made to accommodate a linear cutter stapler and facilitate the removal of the resected specimen. The ipsilateral lung with the PAVM was isolated from ventilation and exposed to atmospheric pressure, allowing for spontaneous lung contraction. When a purple vessel (Fig. 1) or pleural bulge of PAVM was identified, the resection site could be promptly determined. In cases where neither a purple vessel nor pleural bulge was identified, additional efforts were made to locate the PAVM (Figs. 2, 3 and 4). A chest tube was inserted into the thoracic cavity to drain fluids or air after surgery.

Post-surgical complications were evaluated via a general examination conducted by the surgeon, chest X-ray, and blood data analysis.

### Analysis of medical record

The success of VATS was determined by whether or not the venous sac of the PAVM was included in the resected specimen. Descriptions of complications, duration of post-surgical hospital stay (days), duration of chest tube placement (days), VATS time (minutes), and bleeding volume (mL) were evaluated (Table 1).

**Table 1:** Details of patients

case	Age (year)	sex	Sac (mm)	Depth (mm)	Visibility of PAVM	C.T.P (day)	P.S.H.S. (day)	Bleeding Volume (mL)	VATS Time (minute)	note
1	67	M	6.7	0	Visible	4	5	10>	20	
2	78	F	7.7	0	Visible	3	5	10>	25	Note 1)
3	65	F	5.6	0	Visible	3	5	10>	26	
4	64	F	6.4	0	Visible	3	5	10>	34	Note 2)
5	59	F	9.2	0	Visible	2	5	10>	28	
6	65	F	5.1	0	Visible	3	5	10>	52	
7	70	F	6.4	0	Visible	2	3	10>	16	
8	25	M	4.9	0	Visible	3	5	10>	34	
9	62	F	6.2	0	Bulge	3	5	10>	37	
10	40	F	5.8	0	Bulge	3	5	10>	22	
11	56	F	8.1	0	Bulge	2	5	10>	23	
12	75	F	6.1	0	Bulge	3	5	10>	40	
13	71	M	3.5	0	Bulge	3	5	10>	101	
14	60	F	4	0	Bulge	3	5	10>	19	
15	48	F	5.8	0	Bulge	3	10	10>	45	
16	63	F	7.3	0	Bulge	3	4	10>	68	Note 3)
17	55	F	5	1	Bulge	1	5	10>	62	Note 4)
18	40	F	3.7	2.5	Non	1	2	10>	97	Note 5)
19	60	F	4.6	4.6	Non	2	4	10>	24	Note 6)
20	46	M	11.4	5.5	Non	3	5	10>	79	Note 7)
21	62	F	3.9	0	Bulge	3	5	10>	41	Note 8)
			7.8	0	Bulge					
22	60	F	5.2	0	Bulge	3	7	10>	45	Note 9)
23	80	F	9.2	0	Visible	3	5	1900	195	Note 10)
Middle range	55		7.9	5.5		3	8	1900	197	

PAVM: Pulmonary arteriovenous malformation

Depth: The distance between pleural surface/fissure and PAVM

Sac: Diameter of the venous sac of PAVM

C.T.P.: chest tube placement

P.S.H.S.:Postsurgical hospital stay length

Visible: Purple vessel of PAVM was seen through the pleura on thoracoscope

Bulge: Pleural bulge of PAVM was seen immediately after insertion of thoracoscope

Non: Neither purple vessel nor pleural bulge was seen

Note 1) Metachronous resection of lung carcinoma was done

Note 2) Persistent PAVM after transcatheter embolization

Note 3) Metachronous resection of colon carcinoma was done

Note 4) Metachronous resection of colon carcinoma was done

Note 5) Intraoperative ultrasonography was used to identify the PAVM

Note 6) The PAVM was identified by incision of incomplete fissure

Note 7) The bulge of PAVM was identified by excessive spontaneous contraction of lung

Note 8) This patient underwent simultaneous resection of 2 PAVMs and metachronous resection of lung carcinoma

Note 9) Simultaneous wedge resection of lung carcinoma was done

Note 10) Simultaneous lobectomy of lung carcinoma was done

### Analysis of CT images

CT images were used to measure the venous sac diameter of the PAVM and the distance between the pleural surface/fissure and PAVM. The window width was set at 1400 H.U. and the level at -650 H.U.

### Post-surgical CT

Post-surgical CT scans were available for 6 patients who had undergone both PAVM and carcinoma resection. These patients underwent periodic thoraco-abdominal CT scans to check for recurrence and metastases of carcinoma. No post-surgical CT scans were performed for the remaining 17 patients.

## Results

### VATS of PAVM

The venous sac of all 24 PAVMs were included in the resected specimens, and all patients (23/23, 100%) were successfully treated. Only one patient was associated with massive bleeding, and this occurred during simultaneous lobectomy of a lung carcinoma, not during wedge resection of the PAVM. No other serious complications such as death or abscess formation were seen. The length of postsurgical hospital stay, duration of chest tube placement, and VATS time were 5.0 ± 1.4 days (2–10 days), 2.7 ± 0.7 (1–4 days), and 49.3 ± 39.9 min (16–195 min), respectively.

### CT analysis and identification of PAVM

The distance between pleural surface/fissure and PAVM was 0 mm in 20 PAVMs, and 1, 2.5, 4.6, 5.5 mm respectively in the remaining 4. Among the 20 PAVMs with 0 mm distance, the purple vessel of PAVM was visible through the pleura in 9 (Fig. 1) and invisible in 11. In all these 11 invisible PAVMs, the pleural bulge of PAVM was identified soon after insertion of the thoracoscope. In the 4 PAVMs with a distance more than 0 mm, pleural bulge was seen in one with a distance of 1 mm. No purple vessel or pleural bulge was seen in the remaining 3 with a distance of 2.5 mm or more. In these 3 invisible PAVMs without pleural bulge, each PAVM could be identified as follows: one was identified using intraoperative ultrasonography (Fig. 2), one by incision of incomplete fissure (Fig. 3), and the remaining one by standing out the pleural bulge of the PAVM with excessive spontaneous contraction of the lung (Fig. 4).

The venous sac diameter ranged between 3.5 and 11.4 mm (6.2 ± 1.9 mm). No residual PAVM was confirmed in any 6 patients on post-surgical CT.

## Discussion

An important point in our study was the distance between the pleural surface/fissure and PAVM, which could affect the ease of identification during VATS. When the distance was 1 mm or less, purple vessel or pleural bulge of PAVM could be identified in 21 of 21 PAVMs (100%). When the distance was 2.5 mm or more, PAVM could not be identified in any 3 (0%) soon after insertion of thoracoscope and additional efforts were required for identification. If VATS was selected for treatment of PAVM with a distance of 2.5 mm or more, plan and strategy for identification should be prepared such as using intraoperative ultrasonography (Fig. 2).

The mean post-surgical hospital stay was 5 days, and relatively long. This prolonged hospital stay was attributed to the healthcare system in Japan. In Japan, most hospitals provide both early postoperative care and subsequent nursing care within the same hospitalization. Thus, this long stay did not indicate that VATS was so invasive. Nagano et al. analyzed 211 surgical cases on the Japanese Diagnosis Procedure Combination database [12]. Most of cases were treated by VATS, and it was reported that the rate of re-treatment at 2 years was calculated as 2.1% and median hospital stay as 6 days. In our study, the rate of re-treatment was 0/23 (0%) and mean postsurgical hospital stay was 5 days. Both were similar with those of this previous report from Japan.

Previous study [11] showed that the mean VATS time in 6 cases was 50 min (range: 30–95 min). The mean VATS time in our study was 49.3 min and similar with that of this report, and the shortest VATS time was only 16 min. Short VATS time might be brought about by easy and rapid identification of PAVM. Shortness of the distance between pleural surface/fissure and PAVM, visibility of purple PAVM vessel, and largeness of venous sac might be factors for easy identification of PAVM, that might result in easy and safe completion of VATS. However, we could not undertake statistical analysis to examine these factors due to small size of the study population.

In our study, post-surgical CT was done in 6 patients, and no residual PAVM was displayed in any 6 on CT. The rationale for this constraint on the use of post-surgical CT was the following: post-surgical CT was performed to verify recurrence of carcinoma, not to assess PAVM persistence. In reality, the resection’s success could be affirmed by the pathological analysis of the excised specimen, and post-surgical CT was not performed to evade unnecessary irradiation in the remaining 17 patients.

The advantages of VATS over TCE are notable in that there is no persistence of PAVMs after successful resection [12]. This advantage is particularly salient in idiopathic cases, where there is no need for post-surgical imaging after successful treatment since no new PAVMs appear. However, for patients with HHT, follow-up imaging is recommended every 5–10 years after successful treatment to monitor for the development of new PAVMs [5]. Recent advancements in CT and MR imaging technologies have enabled the evaluation of PAVM persistence after TCE without the need for invasive angiography [13, 14], and varying rates of persistence after initially successful TCE have been reported, ranging from 10 to 50% [13–23]. The possibility of PAVM persistence is a significant disadvantage of TCE, as recanalization can occur even after 2 years following initially successful TCE [16]. Persistent PAVMs must be treated as they can lead to in-situ thromboemboli formation [5]. Therefore, all patients should undergo serial follow-up imaging for several years even after initially successful TCE. In a recent retrospective analysis of 378 PAVMs across 8 leading institutions in Japan, persistence was observed in 61 out of 378 cases (16%) after TCE [21]. If a patient with a peripherally located simple-type PAVM desires to avoid serial follow-up imaging examinations after treatment and is willing to accept the invasiveness of VATS, we would recommend VATS as the first-line treatment.

There are several limitations in this study. (1) sample size was so small that no statistical analysis could be done to find factors to select VATS suitable case. (2) All PAVMs in this study were idiopathic simple type. We encountered only 2 patients with complex type PAVM during the study period, and analysis of complex type PAVM was not done.

In conclusion, VATS was safe and effective for treatment of peripherally located simple type PAVM. When the distance between pleural surface/fissure and PAVM was 2.5 mm or more, plan and strategy for identification of PAVM should be prepared before VATS because PAVM may not be easily identifiable and various additional efforts may be required for its identification.

## Data Availability

The datasets used and/or analyzed during the current study are available from the corresponding author on reasonable request.

## References

[1] Shenstone NS (1942). Experience with total pneumonectomy. J Thorac Surg.

[2] Porstmann W, Kelop O (1977). Therapeutic embolization of arteriovenous pulmonary fistula by catheter technique. Current concepts in Pediatric Radiology.

[3] Taylor BG, Cockerrill EM, Manfredi F, Klatte EC (1978). Therapeutic embolization of the pulmonary arteriovenous fistula. Am J Med.

[4] Shovlin CL, Condliffe R, Donaldson JW, Kiely DG, Wort SJ (2017). British thoracic society clinical statement on pulmonary arteriovenous malformations. Thorax.

[5] Majumdar S, McWilliams JP (2020). Approach to pulmonary arteriovenous malformations: a comprehensive update. J Clin Med.

[6] Temes RT, Paramsothy P, Endara SA, Wernly JA (1998). Resection of a solitary pulmonary arteriovenous malformation by video-assisted thoracic surgery. J Thorac Cardiovasc Surg.

[7] Akiyama S, Hanada S, Uruga H, Takaya H, Miyamoto A, Morokawa N (2013). Hereditary hemorrhagic telangiectasia with pulmonary arteriovenous malformations and embolic strokes treated successfully with video-assisted thoracoscopic resection. Intern Med.

[8] Bakhos CT, Wang SC, Rosen JM (2016). Contemporary role of minimally invasive thoracic surgery in the management of pulmonary arteriovenous malformations: report of two cases and review of the literature. J Thorac Dis.

[9] Flury DV, Kocker GJ, Lutz JA, Schmid RA, Dorn P (2020). Uniportal thoracoscopic surgery for pulmonary arteriovenous malformations-report of technique and case series. Curr Chall Thorac Surg.

[10] Reichert M, Kerber S, Alkoudmani I, Bodner J (2015). Management of a solitary pulmonary arteriovenous malformation by video-assisted thoracoscopic surgery and anatomic lingula resection: video and review. Surg Endosc.

[11] Nakajima J, Takamoto S, Takeuchi E, Fukami T, Sano A (2006). Thoracoscopic surgery for pulmonary arteriovenous malformation. Asian Cardiovasc Thorac Ann.

[12] Nagano M, Ichinose J, Sasabuchi Y, Nakajima J, Yasunaga H (2017). Surgery versus percutaneous transcatheter embolization for pulmonary arteriovenous malformation: analysis of a national inpatient database in Japan. J Thorac Cardiovasc Surg.

[13] Kawai T, Shimohira M, Kan H, Hashizume T, Ohta K, Kurosaka K (2014). Feasibility of time-resolved MR angiography for detecting recanalization of pulmonary arteriovenous malformations treated with embolization with platinum coils. J Vasc Interv Radiol.

[14] Remy-Jardin M, Dumont P, Brillet PY, Dupuis P, Duhamel A, Remy J (2006). Pulmonary arteriovenous malformations treated with embolotherapy: helical CT evaluation of long-term effectiveness after 2–21-year follow-up. Radiology.

[15] Letouneau-Guillion L, Faughnan ME, Soulez G, Giroux MF, Oliva VL, Boucher LM (2010). Embolization of pulmonary arteriovenous malformations with Amplatzer vascular plugs: safety and midterm effectiveness. J Vasc Interv Radiol.

[16] Shimohira M, Kawai T, Hashizume T, Ohta K, Nakagawa M, Ozawa Y (2015). Reperfusion rates of pulmonary arteriovenous malformations after coil embolization: evaluation with time-resolved MR angiography or pulmonary angiography. J Vasc Interv Radiol.

[17] Pollak JS, Saluja S, Thabet A, Henderson KJ, Denbow N, White RI (2006). Clinical and anatomic outcomes after embolotherapy of pulmonary arteriovenous malformations. J Vasc Interv Radiol.

[18] Woodward CS, Pyeritz RE, Chittams JL, Trerotola SO (2013). Treated pulmonary arteriovenous malformations: patterns of persistence and associated retreatment success. Radiology.

[19] Adachi A, Ohta K, Jahangiri Y, Matsui Y, Horikawa M, Geeratikun Y (2020). Treatment of pulmonary arteriovenous malformations: clinical experience using different embolization strategies. Jpn J Radiol.

[20] Ratnani R, Sutphin PD, Koshti V, Park H, Chamarthy M, Battaile J (2019). Retrospective comparison of pulmonary arteriovenous malformation embolization with the polytetrafluoroethylene-covered nitinol microvascular plug, Amplatzer plug, and coils in patients with hereditary hemorrhagic telangiectasia. J Vasc Interv Radiol.

[21] Shimohira M, Kiyosue H, Osuga K, Gobara H, Kondo H, Nakazawa T (2021). Location of embolization affects patency after coil embolization for pulmonary arteriovenous malformations: importance of time-resolved magnetic resonance angiography for diagnosis of patency. Eur Radiol.

[22] Kucukay F, Özdemir M, Senol E, Okten S, Ereren M, Karan A (2014). Large pulmonary arteriovenous malformations: long-term results of embolization with Amplatzer vascular plugs. J Vasc Interv Radiol.

[23] Bailey CR, Arun A, Towsley M, Choi WK, Betz JF, MacKenzie S (2019). MVP micro vascular plug systems for the treatment of pulmonary arteriovenous malformations. Cardiovasc Interv Radiol.

